# Supplementary material for: Structural remodeling of target-SNARE protein complexes by NSF enables synaptic transmission
Source: Nat Commun. 2025 Sep 24;16:8371. doi: 10.1038/s41467-025-62764-0 (PMC12460809; doi:10.1038/s41467-025-62764-0)
Supplement: Supplementary file 2 — Description of Additional Supplementary Files [file 41467_2025_62764_MOESM2_ESM.docx]

Title: Supplementary Movie 1

Description: Supplementary Movie 1| A model of NSF motions and substrate translocation associated with nucleotide rebinding and hydrolysis. A sequential hydrolysis model for SNARE complex disassembly by NSF inferred from comparison of different classes from non-hydrolyzing and hydrolyzing conditions. Following hydrolysis-independent substrate side loading, the D1 ring is found in a prehydrolysis state with ATP in protomer E of D1. Protomer F nucleotide exchange then enables ATP binding, reengagement to both SNARE substrate, and the formation of the interprotomer interface with protomer E, triggering ATP hydrolysis in protomer E and substrate translocation. This is accompanied by Pi and Mg2+ release from downring protomers; in many structures of the different classes, this apparent release occurs between protomers C and B. The cycle then begins anew. Blue corresponds to ADP-bound, red to ATP-bound, purple to ADP•Pi bound, and grey to nucleotide-free. See Fig. 10 for key frames and further detail
